# Cross‐cultural adaptation of Nepalese literacy and stigma of suicide scales (LOSS‐SF‐Nep and SOSS‐SF‐Nep) among Nepalese medical and nursing students

**DOI:** 10.1002/brb3.3344

**Published:** 2023-11-27

**Authors:** Anoop Krishna Gupta, Rakhi Sharma, Ram Prakash Sah, Subodh Sharma, Ashish Jha, Manisha Chapagai, Fahimeh Saeed, Sheikh Shoib

**Affiliations:** ^1^ Department of Psychiatry National Medical College Birgunj Nepal; ^2^ Department of Psychiatry National Medical College Birgunj Nepal; ^3^ Department of Psychiatry Institute of Medicine Maharajgunj Nepal; ^4^ Psychosis Research Center University of Social Welfare and Rehabilitation Sciences Tehran Iran; ^5^ Department of Psychiatry Jawaharlal Nehru Memorial Hospital Kashmir India

**Keywords:** LOSS‐SF, Nepal, psychometric properties, SOSS‐SF, stigma, suicide literacy

## Abstract

**Introduction**: Nepal is a country in South‐east Asia with high suicide. There is ongoing trend of emerging research on suicide from Nepal but there is lack of validated scale in measuring literacy or stigma. In the view of poor media reporting and large treatment gap, this study was conducted. All previous validation studies were done in non‐Hindu populations.

**Methods**: A cross‐sectional study was planned where the short forms of Nepalese literacy of suicide scale (LOSS‐SF‐Nep) and Stigma of Suicide Scale (SOSS‐SF‐Nep) were validated using standard procedure at a medical college in southern Nepal. Medical and nursing students of all batches were approached offline after successful pretesting. The psychometric properties of the scales were tested, and the statuses of literacy and stigma were assessed. Patient Health Questionnaire‐9 and General Anxiety Disorder Scale‐7 were used for revealing depression and generalized anxiety.

**Results**: Three hundred and nineteen Nepalese students participated and most of them were males, belonged to nuclear family, upper‐middle socioeconomic status and represented 46 out of 77 districts of Nepal. The mean score of LOSS‐SF‐Nep was 6.36 ± 1.92 and literacy ranged from 37.9% to 89.7%. The deeper exposure to suicidal patients was associated with better literacy. Factor analysis of SOSS‐SF‐Nep revealed three subscales: stigmatization, isolation/depression, and normalization/glorification and had acceptable psychometric properties. Gender, occupation of head of the family, region and years of education, using mental health services, and depression were associated with variable literacy or stigma.

**Conclusion**: Literacy and stigma scales were validated in Nepali, and SOSS factor structures were revealed with modified descriptors. The literacy and stigma levels in medical students were calculated for the first time in Nepal and Hindu majority population.

## Abstract 2

1.Literacy and stigma scales were validated in Nepali and the SOSS factor structure was revealed with modified descriptors. 2.The literacy and stigma levels in medical students were calculated for the first time in Nepal and the Hindu majority population.

## 1 INTRODUCTION

The age‐standardized suicide rate in South‐East Asia is higher than the global average (Marahatta et al., 2017; Thapaliya et al., 2018). Nepal is a South Asian country with about 13% prevalence of mental disorders and a high rate of substance use disorders, dissociative conversion disorder, and major depressive disorder that are known as risks of suicide (Jha et al., 2019; Rai et al., 2020). Its age‐standardized suicide rate for both sexes in 2019 was 9.8 per 100,000 people (Organization, 2021), and the suicide rate in Nepalese adolescents is 10.33% (Pandey et al., 2019). Despite the gravity of the situation, suicide is taboo, and suicide attempt is a crime that leads to stigmatization and harsh media reporting (Sharma et al., 2022; Thapa et al., 2021; Utyasheva et al., 2022).

Among several factors that are related to suicide, mental disorders are predominant. The stigma of mental disorders and suicide in low–middle‐income countries like Nepal is high, which leads to less help‐seeking behavior (Kudva et al., 2020; Shoib et al., 2022). Stigmatization, social isolation, discrimination, self‐blame, and negative beliefs about psychiatric disorders are obstacles for suicidal individuals to seek help (Carpiniello & Pinna, 2017). In Nepal, criminalization of suicide, underreporting suicide and avoiding its documentation, unwilling reporting suicide by family and suicidal people as a result of stigma and legal entanglements, and financial disadvantages are barriers to suicide prevention strategies (Hagaman et al., 2016).

The first step to reducing stigma is increasing society's knowledge and attitude toward suicide (Niederkrotenthaler et al., 2014). About 75% of the victims of suicide visited primary care providers within the year of leading to suicide, and near about 50% of them did in the month before (Luoma et al., 2002) which can act as opportunities to prevent. If the victims are destigmatized and decriminalized, they can be saved during such interactions. Additionally, studies showed that low level of Literacy about suicide is not limited to general population, and it also involves Nepalese health professionals (Shah et al., 2022). So, there is a need for a study measuring literacy and stigma in Nepalese population in order to evaluate the severity of the problem and cultural differences from other studied populations.

Till date, there is no culturally adapted instrument to measure literacy or stigma of suicide in Nepali language. Attitudes toward suicide and literacy have been studied locally but with scales in English (Shah et al., 2022; Thapa et al., 2021). There are two scales, namely, literacy of suicide scale (LOSS) and Stigma of Suicide Scale (SOSS) that have been validated cross‐culturally in Australia, China, Turkey, Middle East, and Bangladesh (Al‐Shannaq & Aldalaykeh, 2021; Arafat et al., 2022; Batterham et al., 2013b, 2021; Calear et al., 2012; Chan et al., 2014; Han et al., 2017). Therefore, we aimed to adapt and psychometrically validate the LOSS‐short form (LOSS‐SF) and SOSS‐short form (SOSS‐SF) in Nepali and subsequently assess the level of stigma and literacy of suicide among the medical students of Nepal. This eventually will help formulate national suicide prevention and education plan.

## 2 METHODOLOGY

### 2.1 Study site, samples, and procedures

This was a descriptive, cross‐sectional study at National medical college, Birgunj, Nepal. Students from Nepal and India come to pursue medical and nursing degrees at this tertiary care center. Nepali speaking students from six batches including interns were approached through purposive sampling. Printed questionnaires were provided manually that took around 15–20 min to fill. With an aim to reach only Nepalese students in college premises, 340 students above 18 years could be approached and 319 had completed responses. They belonged to 46 districts that represented all 3 topographical regions (terai, hills, and mountains) of Nepal. Data were collected between 1 and 30 November, 2022. The principal author had access to all data and sociodemographic profile was separated from the scales while coding to maintain anonymity. Psychiatric help was offered to participants through emails and phone calls by the principal author.

### 2.2 Adaptation of LOSS‐SF and SOSS‐SF in Nepali (LOSS‐SF‐Nep and SOSS‐SF‐Nep)

Beaton et al. (2000) was followed for the validation of LOSS and SOSS. Two bilingual experts (a psychologist and a psychiatric resident) were involved in the translation, and they faced hurdle while translating the words, “diagnosis” and “psychosis.” The literal meaning was discarded in view of adapting for local acceptance that meant “finding out the disease” and “mad/severe mental illness.” Similarly, two words were preferred to single word descriptors in SOSS like “pathetic” and “shallow.” This was done for wider acceptance in Nepal which has multiple versions (dialects) of the Nepali language. Other disparities were resolved when shorter sentences were preferred and a common version was generated after synthesis. The other two volunteers (a psychiatrist and a dentist) were involved in back translation. They used synonymous words for “depression” and a common word was agreed upon during expert committee meeting that involved the principal author. Meanwhile, they used same word for “lost” and “lonely.” So, three‐word descriptor was used for “lonely” and two‐word one for “lost.”

A total of 32 interns, nursing students, and nonmedical volunteers were involved as pilot samples. This sample was not included in the analysis group of 319 study sample. They filled sociodemographic questionnaire, Patient Health Questionnaire‐9 (PHQ‐9), and General Anxiety Disorder Scale‐7 (GAD‐7) those have been used in Nepali before (Gupta, Mehra, et al., 2020; Gupta, Sahoo, et al., 2020). We consider score of 10 to diagnosis depression by PHQ‐9 and anxiety by GAD‐7. All were interviewed for their understanding and difficulty of the final Nepali version. Minor edits were done in the final adaptation process. Thus, self‐reported LOSS‐Nep and SOSS‐Nep were determined as the final version of the scales.

### 2.3 Instruments

#### 2.3.1 Sociodemographic questionnaire

A self‐designed, semi‐structured questionnaire was used to collect sociodemographic profile. It included age, gender, religion, locality, academic year, marital and family status, history of psychiatric disorders and suicide behaviors, and level of exposure to suicide behavior in family or society. Suicide exposure was assessed through graded form.

#### 2.3.2 Nepalese literacy of suicide scale‐short form (LOSS‐SF‐Nep)

It was adapted from Calear et al. (2012) whose short version has 12 items (Aldalaykeh et al., 2020; Arafat et al., 2022). All sentences had three options as “yes,” “no,” and “don't know.” A total score was generated between 0 and 12 that was converted as percentage. It evaluates the suicide‐related literacy under three themes of risk factors (three items), signs/symptoms (three items), cause (four items), and prevention/treatment (two items). The internal consistency was not justified due to response pattern of the scale. The scale has 2, 4, 6, 8, and 11 as true statements.

#### 2.3.3 Nepalese stigma of suicide scale‐short form (SOSS‐SF‐Nep)

It was adapted from Batterham et al. (2013b). This 16‐item scale has one‐ to three‐word descriptor, and its response was rated on five‐point Likert scale from strongly disagree (1) to strongly agree (5). It assesses stigmatization (eight items), glorification (four items), and isolation (four items) which form three subscales. The internal consistencies of these subscales were .67, .72, and .64, respectively, using Cronbach's alpha coefficient.

### 2.4 Depression and anxiety scales

Nepali versions of PHQ‐9 and GAD‐7 scales were used to assess any depression and generalized anxiety, respectively (Gupta, Mehra et al., 2020; Gupta, Sahoo et al., 2020).

### 2.5 Data analysis

The statistical package for the social sciences (SPSS) version 23 was used for analysis. The sociodemographic variables, LOSS‐SF‐Nep, and SOSS‐SF‐Nep scores were expressed in percentage, mean and standard deviation and their mean scores were compared using *t*‐test. The internal consistency (reliability) was measured using Cronbach's alpha coefficient and >.6 was the acceptable limit. The face and content validity were determined during the adaptation process. For this, six experts from psychology, nursing, and nonpsychiatric faculties from the college and outside were asked to volunteer. They agreed upon all items of LOSS to be measuring literacy but questioned if “shallow” could be associated with a suicidal person. Each item from LOSS and SOSS was voted for being “essential,” “useful,” or “unnecessary.” They voted all items of LOSS to be “essential.” For SOSS, they voted “dedicated” and “shallow” as useful but not essential and rest to be essential items. Factor analysis revealed the construct validity and was done using varimax rotation with Kaiser Normalization, Scree Plot analysis with fixed three factors.

### 2.6 Ethical clearance

Permissions were taken from the instrument developing authors (Batterham et al., 2013b) and Institutional Review Board of National Medical College (Ref. F‐NMC/534/077‐078) on June 25, 2021. All participants were given contact details of our treating psychiatrist in the department for indicated referrals. Written consents were taken after explaining the study design to all participants.

## 3 RESULTS

All participants were medical and nursing students between 18 and 27 years of age from several batches. All except one were single, and 54% were males. The participants belonged to 47 out of 77 districts in Nepal that ranged from southern plains to northern mountains.

### 3.1 LOSS‐SF‐Nep

Percentages of correct responses to LOSS are depicted in Table 1 where they have more difficulty in recognizing the signs/symptoms and had ease with treatment/prevention of suicide. In average, 55% of participants gave correct response for all 12 items.

**TABLE 1:** Percentage correct responses to items of Nepalese literacy of suicide scale (LOSS‐SF‐Nep).

Item number	Item	Percentage correct answer (*n* = 319)	Literacy theme
LOSS_2	Seeing a psychiatrist or psychologist can help prevent someone from suicide (T)	89.7	Treatment/prevention
LOSS_9	People who have thoughts about suicide should not tell others about it (F)	78.7	Treatment/prevention
LOSS_3	Most people who suicide are psychotic (F)	77.4	Risk factor
LOSS_8	Not all people who attempt suicide plan their attempt in advance (T)	63.6	Sign/symptom
LOSS_4	There is a strong relationship between alcoholism and suicide (T)	55.2	Risk factor
LOSS_7	Talking about suicide always increases the risk of suicide (F)	53.9	Cause/nature
LOSS_10	Very few people have thoughts about suicide (F)	48.3	Cause/nature
LOSS_1	If assessed by a psychiatrist, everyone who suicides would be diagnosed as depressed (F)	42.3	Cause/nature
LOSS_11	Men are more likely to suicide than women (F)	39.8	Risk factor
LOSS_5	People who talk about suicide rarely kill themselves (F)	39.5	Sign/symptom
LOSS_12	A suicidal person will always be suicidal and entertain thoughts of suicide (F)	39.5	Cause/nature
LOSS_6	People who want to attempt suicide can change their mind quickly (T)	37.9	Sign/symptom

Abbreviations: F, false; T, true.

### 3.2 SOSS‐SF‐Nep

Table 2 shows the responses and factor loadings of SOSS‐Nep. Maximum agreements (63.6%–86.3%) were seen with isolation section, whereas poor scores were seen with the stigmatizing descriptors. Factor rotation revealed 42% (16.0, 14.6, and 11.3) of cumulative variance. Five items (an embarrassment, vengeful, pathetic, disconnected, and dedicated) had loadings less than 0.5, and thus, they were dropped. The final SOSS‐Nep contains 11 items: stigmatization (irresponsible, stupid, coward, immoral, and shallow); isolation (lonely, isolated, and lost); and normalization (noble, strong, and brave).

**TABLE 2:** Responses and factor loadings for items of Nepalese stigma of suicide scale (SOSS‐SF‐Nep).

Item	Agree/strongly agree (%)	SOSS‐Nep score Mean (SD)	Factor loadings		
			Stigmatization	Isolation/depression	Normalization/glorification
Irresponsible	29.8	2.74 (1.21)	**.638**	.083	−.105
Stupid	33.6	2.90 (1.22)	**.626**	.040	−.163
Coward	32.9	2.86 (1.18)	**.625**	.036	−.255
Immoral	16.3	2.45 (1.03)	**.600**	.155	−.157
Shallow	20.0	2.79 (.95)	**.586**	.096	.087
An embarrassment	43.2	3.06 (1.27)	.472	.232	.034
Vengeful	16.9	2.39 (1.08)	.424	−.115	.172
Pathetic	33.9	2.90 (1.13)	.342	.159	.256
Disconnected	63.6	3.51 (1.22)	.289	.443	.159
Lonely	86.3	4.15 (.99)	.107	**.858**	−.045
Isolated	73.0	3.82 (1.06)	.097	**.767**	−.026
Dedicated	25.4	2.68 (1.07)	.058	−.139	.445
Lost	80.5	3.98 (1.00)	.041	**.780**	−.014
Noble	6.9	1.98 (1.00)	−.007	−.079	**.609**
Strong	16.9	2.38 (1.09)	−.141	.178	**.678**
Brave	20.6	2.37 (1.23)	−.151	.144	**.727**

*Note*: Bold values indicate factor loadings above .5.

Abbreviation: SOSS‐Nep, Nepalese Stigma of Suicide Scale.

### 3.3 Demographic, psychiatry history, and exposure to suicide factors

Table 3 reveals that females had better literacy and lesser stigma than in Bangladesh and Australia, though nonsignificant (Arafat et al., 2022; Batterham et al., 2013b). Male and female participants had significantly higher stigmatization and normalization, respectively. Exposure to suicide was related to higher literacy. The mean of LOSS‐Nep score was 6.36 ± 1.92, 18.5% had score of 7.0, the median score was 6.0, and the range was 11. The score range of 1–6 was seen in 51%. Suicide attempters had higher LOSS score of 7.17 ± 2.71. Participants with unemployed or retired guardians had higher literacy and lower isolation score. People from plains (southern Nepal) had lower literacy and higher isolation score. Knowing someone who had died by suicide and having suicidal ideation were associated with higher literacy and later was associated with normalization. Senior students and people who have used mental health service had better literacy, whereas the later had lesser stigma also. Having current depression was associated with normalization. Around 18% of the students had clinical depression, whereas 14% had generalized anxiety. Having depression was significantly associated with higher normalization but not with literacy, stigma, or isolation of suicide.

**TABLE 3:** Descriptive statistics for the study samples and relationship with literacy of suicide scale (LOSS‐SF‐Nep) and stigma of suicide scale (SOSS‐SF‐Nep).

Variable	Category	*N* (%)	LOSS‐SF‐Nep Mean (SD)	Stigmatization Mean (SD)	Isolation/depression Mean (SD)	Normalization/glorification mean (SD)
Gender	Male Female	171 (53.61) 148 (46.39)	6.19 (2.0) 6.55 (1.82)	2.91 (.61)*** 2.59 (.61)	3.88 (.81) 3.86 (.76)	2.23 (.73) 2.49 (.67)**
Religion	Hindu Others	283 (88.70) 36 (11.29)	6.39 (1.93) 6.14 (1.88)	2.75 (.63) 2.89 (.59)	3.85 (.81) 3.98 (.59)	2.33 (.71) 2.47 (.71)
Education of head of family	<Higher school High school graduate >Higher school	56 (17.55) 98 (30.72) 165 (51.72)	6.39 (1.66) 6.08 (1.87) 6.52 (2.03)	2.70 (.58) 2.79 (.62) 2.77 (.65)	3.81 (.79) 3.84 (.76) 3.90 (.81)	2.42 (.86) 2.34 (.60) 2.33 (.72)
Occupation of head of family	Full time/part time Unemployed/retired	290 (90.91) 29 (9.09)	6.29 (1.91)* 7.07 (1.94)	2.76 (.61) 2.75 (.82)	3.90 (.77) 3.60 (.92)*	2.36 (.70) 2.30 (.83)
Family income	<50 k 50−100 k >100 k Missing data	104 (32.60) 74 (23.20) 27 (8.46) 114 (35.74)	6.19 (1.84) 6.72 (2.12) 7.00 (2.08) NA	2.80 (.64) 2.85 (.69) 2.69 (.49)	3.80 (.89) 4.03 (.81) 4.16 (.66)	2.31 (.76) 2.35 (.71) 2.29 (.55)
Family type	Nuclear Joint Extended	228 (71.5) 67 (21.0) 24 (7.5)	6.36 (1.87) 6.09 (2.07) 7.08 (1.91)	2.76 (.62) 2.81 (.67) 2.65 (.63)	3.90 (.76) 3.76 (.92) 3.86 (.63)	2.35 (.70) 2.35 (.77) 2.31 (.70)
Locality	Rural Urban	118 (37.0) 201 (63.0)	6.26 (1.82) 6.42 (1.98)	2.82 (.67) 2.73 (.60)	3.85 (.77) 3.88 (.80)	2.36 (.68) 2.34 (.73)
Region/terrain	Plains Hilly/mountainous	226 (70.85) 93 (29.15)	6.22 (1.94)* 6.71 (1.84)	2.79 (.64) 2.70 (.60)	3.80 (.80) 4.02 (.74)*	2.31 (.71) 2.46 (.71)
Family history of mental illness	Present Absent	37 (11.59) 282 (88.40)	6.89 (1.52) 6.29 (1.96)	2.74 (.66) 2.76 (.62)	4.05 (.55) 3.84 (.81)	2.40 (.74) 2.34 (.71)
Family history of suicidal attempt	Yes No	16 (5.01) 303 (94.98)	7.00 (1.62) 6.33 (1.93)	2.59 (.63) 2.77 (.63)	3.97 (.97) 3.86 (.78)	2.50 (.59) 2.34 (.72)
Any known person died by suicide	Yes No	148 (46.4) 171 (53.6)	6.64 (1.88) 6.12 (1.93)*	2.71 (.65) 2.81 (.61)	3.92 (.75) 3.82 (.82)	2.35 (.70) 2.35 (.73)
Suicidal ideation, lifetime	Yes No	70 (21.9) 249 (78.1)	6.84 (2.05) 6.22 (1.87)*	2.64 (.66) 2.80 (.61)	4.01 (.81) 3.82 (.78)	2.50 (.64)* 2.31 (.73)
Batch (academic year)	First year Second year Third year Forth year Interns	93 (29.2) 60 (18.8) 74 (23.2) 68 (21.3) 24 (7.5)	5.55 (1.98)*** 6.48 (1.82) 6.45 (1.78) 6.93 (1.84) 7.33 (1.58)	2.85 (.68) 2.73 (.66) 2.83 (.57) 2.65 (.57) 2.57 (.63)	3.83 (.72) 3.94 (.74) 3.86 (.98) 3.90 (.68) 3.73 (.79)	2.46 (.70) 2.34 (.79) 2.22 (.67) 2.33 (.70) 2.42 (.68)
Ever used mental health services	Yes No	37 (11.6) 282 (88.4)	7.16 (1.92) 6.26 (1.90)**	2.54 (.69) 2.79 (.61)*	3.70 (.98) 3.89 (.76)	2.43 (.79) 2.34 (.70)
Exposure to suicide	No exposure at all	33 (10.3)	5.64 (1.98)	2.88 (.65)	3.75 (.72)	2.21 (.74)
	Observing suicide in movie or television	132 (41.4)	6.28 (1.96)	2.82 (.54)	3.90 (.72)	2.30 (.71)
	Watched a documentary on suicide	40 (12.5)	6.6 (1.97)	2.75 (.49)	4.13 (.76)	2.44 (.73)
	Colleague/s attempted or died by suicide	21 (6.6)	6.14 (2.22)	2.74 (.64)	3.62 (1.09)	2.25 (.72)
	Ever provided service to someone who attempted/died by suicide	7 (2.2)	7.14 (2.12)	2.80 (.94)	3.82 (.35)	2.50 (.69)
	Acquaintance/s attempted/died by suicide	8 (2.5)	6.75 (1.16)	2.19 (.68)	3.53 (.90)	2.59 (.82)
	Relative/s attempted/died by suicide	46 (14.4)	6.76 (1.75)	2.73 (.76)	3.94 (.70)	2.35 (.71)
	Close friend/s attempted/died by suicide	16 (5.0)	6.38 (1.45)	2.78 (.62)	3.83 (1.04)	2.44 (.62)
	Ever lived with someone who attempted/died by suicide	10 (3.1)	6.10 (1.29)	2.45 (.57)	3.25 (1.09)	2.68 (.79)
	You ever attempted suicide	6 (1.9)	7.17 (2.71)	2.33 (1.11)	4.04 (.56)	2.54 (.51)
PHQ‐9 depression	Yes (score ≥ 10) No (score < 10)	57 (17.87) 262 (82.13)	6.14 (1.86) 6.41 (1.94)	2.80 (.64) 2.75 (.63)	3.91 (.78) 3.86 (.79)	2.53 (.60)* 2.31 (.73)
GAD‐7 generalized anxiety	Yes (score ≥ 10) No (score < 10)	43 (13.48) 276 (86.52)	6.33 (1.95) 6.37 (1.92)	2.77 (.63) 2.76 (.63)	3.83 (.79) 3.87 (.79)	2.41 (.70) 2.34 (.72)

Abbreviation: NA, not available.

*p < .05, **p < .01, ***p < .001.

Correlation study revealed that stigma and isolation were positively correlated (*p* < .01) as seen in Table 4.

**TABLE 4:** Correlation between literacy of suicide scale (LOSS‐SF‐Nep) and stigma of suicide scale (SOSS‐SF‐Nep).

		Correlations			
		LOSS‐SF‐Nep score	Stigmatization score of SOSS‐SF‐Nep	Isolation/depression score of SOSS‐SF‐Nep	Normalization/glorification score of SOSS‐SF‐Nep
LOSS‐SF‐Nep	Pearson Correlation	1	−.201^a^	−.036	.072
	Sig. (2‐tailed)		.000	.521	.198
	N	319	319	319	319
Stigmatization score of SOSS‐SF‐Nep	Pearson Correlation	−.201^a^	1	.305^a^	−0.138^b^
	Sig. (2‐tailed)	.000		.000	.014
	N	319	319	319	319
Isolation/depression score of SOSS‐SF‐Nep	Pearson Correlation	−.036	.305^a^	1	.051
	Sig. (2‐tailed)	.521	.000		.359
	N	319	319	319	319
Normalization/glorification score of SOSS‐SF‐Nep	Pearson Correlation	.072	−.138^b^	.051	1
	Sig. (2‐tailed)	.198	.014	.359	
	N	319	319	319	319

^a^ Correlation is significant at the .01 level (2‐tailed).

^b^ Correlation is significant at the .05 level (2‐tailed).

## 4 DISCUSSION

This was the first Nepalese study to test the psychometric properties of LOSS‐SF and SOSS‐SF in Nepali language among adult subjects to our knowledge. Previous Asian validation studies were done among Muslim and Chinese population, and thus, this study gives a new perspective from the Hindu majority population. Only 319 samples were possible but they represented varying cultures from all three terrains of Nepal. Previous validation was done among 160, 529, and 224 student samples (Aldalaykeh et al., 2020; Arafat et al., 2022; Han et al., 2017).

Previous Nepalese studies report lack of education and poverty being associated with suicidal ideation (Garrison‐Desany et al., 2020; Hagaman et al., 2018) but current study showed the suicidality in 22% of well‐educated medical students from upper‐middle socioeconomic background. The possible causes of high prevalent suicidal ideation among medical students could be substance use, relationship issues, violence, and singlehood as found in eastern Nepal (Bhattachan et al., 2021). As our samples were biased for education, this finding needs further validation.

Exposure to suicide has been studied in comparison to literacy and stigma which cannot provide causality. These preliminary findings, however, guide toward the need for detailed assessment like degree of relatives attempting suicide or intent and lethality of own suicidal experience. Higher score of isolation among those who saw documentary on suicide is possibly because suicide is generally associated with depression/isolation on screen.

This study revealed higher suicide literacy in comparison to previous studies from Jordan, Bangladesh, and India (Al‐Shannaq & Aldalaykeh, 2021; Aldalaykeh et al., 2020; Arafat et al., 2022; Ram et al., 2017). This could be expected from the medical students. Shah et al. (2022) found a similar literacy among the Nepalese medical professionals in Chitwan, central Nepal. In this study, in average, 55% of participants gave correct response for all 12 items of LOSS‐SF. However, Shah et al. (2022) used 26‐item LOSS and the average of correct response of the same 12 items was 52% in our study. Despite using English version, the results were similar because of educated study population (Shah et al., 2022). In this study, most of the respondents believed that seeing a psychiatrist or psychologist can help to prevent suicide. However, the knowledge about the cause, nature, signs, and symptoms is lacking among them. They need to be emphasized in future educational and anti‐stigma campaigns.

Dropping five items (an embarrassment, vengeful, pathetic, disconnected, and dedicated) reveals the attitude of Nepalese population toward suicide and similarity to other Asian studies (Arafat et al., 2022; Han et al., 2017). These items did not represent the same construct in this culture. Nepalese culture seems unlikely to accept a suicidal person as “vengeful” or “dedicated” as the earlier one is expected to harm others and the later is likely to be hopeful for tangible goal or future achievement. Being dedicated is unlikely to be attached to positive connotation while being “determined” would better describe a suicidal attitude in this culture. The participating responders declined “an embarrassment” as a descriptor as it defies medical or disease model. Factor loading of “disconnected” (0.44) could cross the cut off with larger sample size and needs to be further assessed. The three factors of stigmatization, isolation, and normalization have been as same as original Australian and other Asian studies (Arafat et al., 2022; Batterham et al., 2013a).

The positive correlation of stigma and isolation indicates the participants’ attitude toward suicide which was also seen in Asian cultures (Aldalaykeh et al., 2020; Arafat et al., 2016) but not in Australian study (Batterham et al., 2013a). Similarly, the isolation subscale had higher loading followed by stigma and normalization. Thus, the normalization stands out and is not correlated in Nepalese culture. These findings raise concern about the scale modification for current population.

Low literacy and high stigma in males and the plains/terai region (southern Nepal) demand for educational and destigmatization programs in these groups. Exposure to suicidal patients, mental health services, psychiatric lectures and clinical rounds indicate the benefits of compulsory psychiatric posting for students and interns. Thus, lobbying to separate the psychiatric subject from internal medicine may enhance literacy among students.

The literacy and stigma did not vary significantly in depression or anxiety except in normalizing suicide among those with clinical depression. This normalization was also seen in previous study (Batterham et al., 2013a). Normalizing suicide is an ominous sign, which can be checked in clinical or community scenario by assessing depression.

### 4.1 Implications

The major implication of this study was the validation and reliability of LOSS‐SF‐Nep and SOSS‐SF‐Nep in the Nepalese population for its widespread application. The stigmatizing attitudes and level of literacy can be further tested among news reporters, police officials, and teachers. This can help plan intervention strategies like psychoeducation and school education programs. Less stigmatization and more literacy in females may imply that they can be deployed as anti‐suicide counselors, campaigners, and educators.

### 4.2 Future directions

Larger community samples and test–retest reliability would bolster the wide acceptability of these scales. Depression stigma scales can be applied to further validate the findings. The application of full form of LOSS (26 items) and SOSS (58 items) could strengthen the factor coherence in future samples.

### 4.3 Limitations

Only internal consistency test of reliability was done, and the detailed psychometric properties of LOSS‐Nep are not possible because of the edumetric nature of the scale. This study was limited to college students and thus lacks generalizability for community population. Further, alpha was set low and could be improvised in studies with larger and diverse samples.

## 5 CONCLUSION

The short forms of literacy and stigma scales were validated in Nepali, and stigmas of suicide factor structure were revealed with modified descriptors. The literacy and stigma levels in medical students were calculated for the first time in Nepal and Hindu majority population.

## AUTHOR CONTRIBUTIONS

**Anoop Krishna Gupta**: Conceptualization; data curation; formal analysis; writing—original draft. **Rakhi Sharma**: Data curation; resources; visualization. **Ram Prakash Sah**: Methodology; validation; writing—review and editing. **Subodh Sharma**: Data curation; investigation; software. **Ashish Jha**: Project administration; resources. **Manisha Chapagai**: Supervision; writing—review and editing. **Fahimeh Saeed**: Resources; supervision; writing—review and editing. **Sheikh Shoib**: Supervision; writing—review and editing.

## CONFLICT OF INTEREST STATEMENT

The authors declare no conflicts of interest.

### PEER REVIEW

The peer review history for this article is available at https://publons.com/publon/10.1002/brb3.3344.

## Data Availability

The data that support the findings of this study are available from the corresponding author upon reasonable request.
